# Gender differences in clinical, laboratory and polysomnographic features of restless legs syndrome

**DOI:** 10.1111/jsr.12875

**Published:** 2019-06-04

**Authors:** Evi Holzknecht, Margarethe Hochleitner, Gregor K. Wenning, Birgit Högl, Ambra Stefani

**Affiliations:** ^1^ Department of Neurology Medical University of Innsbruck Innsbruck Austria; ^2^ Gender Medicine Unit Medical University of Innsbruck Innsbruck Austria

**Keywords:** Periodic leg movements during sleep, polysomnography, RLS phenotype, RLS severity scales, symptoms manifestation

## Abstract

Restless legs syndrome is a common neurological disorder with a clear female predominance. This study aims to evaluate gender differences in clinical, laboratory and polysomnographic features in patients with restless legs syndrome. For this retrospective analysis, 42 women and 42 men from the Innsbruck RLS database matched by age and therapy were included. Demographic data as well as different severity scales (IRLS, RLS‐6 and CGI) were evaluated. Laboratory parameters included several indicators of serum iron status. In all patients, polysomnography was performed according to the AASM guidelines, and periodic leg movements during sleep were scored according to the AASM criteria. IRLS, RLS‐6 and CGI revealed more severe symptoms in women (IRLS median [range]: 17.5 [0–35] versus 13.5 [0–32], *p* = 0.028; RLS‐6 median [range]: 18 [0–39] versus 12 [1–42], *p* = 0.014). Women had lower serum ferritin levels than men (median [range] in μg L^−1^: 74 [9–346] versus 167 [15–389], *p* < 0.001). Twenty‐two women and eight men (53.7% versus 22.2%, *p* = 0.003) had ferritin values below 75 μg L^−1^. Periodic leg movements during sleep indices were significantly lower in women than in men (median [range] in number per hr: 11.4 [0–62.5] versus 40 [0–154], *p* = 0.004, and 12.6 [0–58.5] versus 40 [0.5–208], *p* = 0.002, for night I and night II, respectively). Restless legs syndrome severity as measured by validated scales was worse in women, while periodic leg movements during sleep indices were higher in men. These results suggest a possible gender difference in phenotypical presentation of restless legs syndrome, manifesting with predominantly sensory symptoms in women and predominantly motor symptoms in men.

## 1 INTRODUCTION

Restless legs syndrome (RLS) is a common sensorimotor disorder characterized by an urge to move the legs, often accompanied by uncomfortable sensations that begin or worsen at rest, and are partially or totally relieved by movement. Symptoms only occur or worsen in the evening or at night (Allen et al., 2014). Community‐based studies indicate a prevalence of up to 10% in the European and North American populations (Berger, Luedemann, Trenkwalder, John, & Kessler, 2004; Hening et al., 2004; Hogl et al., 2005), with 1.6%–3.4% requiring or seeking treatment (Happe, Vennemann, Evers, & Berger, 2008; Hening et al., 2004).

According to several studies, reported prevalence in women is about twice as high as in men (Berger et al., 2004; Hening et al., 2004; Hogl et al., 2005). Pregnancy and parity represent risk factors for RLS, thus contributing to the higher prevalence of RLS in women (Berger et al., 2004; Manconi et al., 2012).

Little is known about gender differences in the phenotypical presentation of RLS. A relationship between female sex and earlier age of onset has been described, and an influence of environmental (i.e. hormonal) and mitochondrial genetic factors was suggested (Hanson et al., 2004). A questionnaire‐based analysis on clinical RLS presentation reported that the majority of patients with the combination of involuntary movements while awake, sleep‐onset difficulties, and standing up in the night to walk, was female. Additionally, both male and female subjects were more aware of female relatives affected by RLS than of male ones (Bentley, Rosman, & Mitchell, 2006). On the other side, studies about health‐related quality of life in RLS did not show any differences between men and women (Abetz et al., 2004; Happe et al., 2009).

Periodic leg movements during sleep (PLMS) do appear in different sleep disorders and in the general population, but they are present in more than 80% of patients with RLS (Montplaisir et al., 1997; Moore et al., 2014). Gender does not seem to affect PLMS index in healthy sleepers (Frauscher et al., 2014). However, in epidemiological studies a higher prevalence of PLMS index > 15 per hr in men has been reported in European populations (Haba‐Rubio et al., 2016; Szentkiralyi et al., 2019). Only a few studies investigated gender differences in PLMS indices in patients with RLS, and reported controversial results (Montplaisir et al., 1997; Nicolas, Michaud, Lavigne, & Montplaisir, 1999; Shin et al., 2016).

As different factors influence the pathogenesis and clinical manifestation of RLS, probably including gender‐specific factors, we hypothesized that RLS would manifest with different phenotypic features in men and women. The aim of this study was to evaluate the presence of gender differences in clinical, laboratory and polysomnographic features in patients with RLS.

## 2 METHODS

### 2.1 Patient selection

Forty‐two women and 42 men were selected from the Innsbruck RLS database, of the Sleep Disorder Unit, Department of Neurology, Medical University of Innsbruck. Only patients who underwent polysomnography (PSG) were selected. As more men than women underwent PSG, for reasons that have been previously reported (Auer, Frauscher, Hochleitner, & Hogl, 2018), we first included 42 consecutive women. They were then matched for age (± 5 years) and therapy group (untreated, dopaminergic therapy, α2δ‐ligands, and combination therapy) with 42 men. Patients under any other therapy with possible influence on RLS (e.g. benzodiazepines, neuroleptics and antidepressants), patients with iron substitution in the previous 6 months, and patients with a C‐reactive protein (CRP) ≥ 0.5 mg dl^−1^ were not included.

This study was approved by the Ethics Committee of the Medical University of Innsbruck.

### 2.2 Clinical evaluation

Restless legs syndrome was diagnosed according to the current International RLS Study Group (IRLSSG) criteria (Allen et al., 2014). All patients were evaluated by a neurologist expert in sleep medicine, who collected the medical history (including co‐morbidities related to RLS), excluded mimics, and performed a clinical evaluation. The following validated severity scales were completed: IRLSSG severity scale (IRLS) (Walters et al., 2003); RLS‐6 (Kohnen et al., 2016); and the Clinical Global Impression (CGI; National Institute of Global Health, 1976). Current RLS medication was recorded and, in patients receiving dopaminergic therapy, levodopa equivalent dose (LED) and LED per kg were calculated (Tomlinson et al., 2010). Current augmentation was evaluated according to the Max Planck Institute criteria (Garcia‐Borreguero et al., 2007). Information about family history of RLS was collected. Age at RLS onset was documented, and patients were classified in early (first symptoms appearing at the age of ≤ 45 years) or late onset (Allen, La Buda, Becker, & Earley, 2002; Earley, Barker, Horská, & Allen, 2006). In addition to the co‐morbidities reported at the time of the visit to the Sleep Disorder Unit, patients’ records were retrospectively checked for the following conditions possibly aggravating PLMS: rapid eye movement (REM) sleep behaviour disorder (RBD), congestive heart failure, spinal cord injuries, sleep‐related eating disorder, L‐dopa‐responsive dystonia, sickle cell disease, post‐traumatic stress disorder, Asperger's syndrome, William's syndrome, and alcohol dependency (American Academy of Sleep Medicine, 2014; Hornyak, Feige, Riemann, & Voderholzer, 2006).

### 2.3 Laboratory parameters

Laboratory parameters included serum iron, ferritin, transferrin, transferrin saturation, soluble transferrin receptor and CRP. For comparison of iron parameters, all patients with ferritin values ≥ 400 μg L^−1^ and transferrin saturation ≥ 45% were excluded and referred to further haematological examination. The other patients were stratified in two groups, with serum ferritin values of ≤ 75 and > 75 μg L^−1^.

### 2.4 Polysomnography

For the evaluation of PSG parameters, two nights were selected: the first night of PSG (night I), and the subsequent night with the most adequate positive airway pressure therapy, if necessary (night II). PSG was performed according to the American Academy of Sleep Medicine (AASM) criteria, and consisted of electrooculography, electroencephalography (F3, F4, C3, C4, O1, O2, M1 and M2 electrodes), cardiorespiratory recording (single‐channel electrocardiography, recording of nasal air flow [thermocouple], nasal pressure cannula, tracheal microphone, thoracic and abdominal respiratory movements [piezo], transcutaneous oxygen saturation), electromyography including at least the mental, submental and both anterior tibialis muscles and time‐synchronized digital videography with an infrared camera. Leg movements were recorded according to the AASM criteria, using surface electrodes placed longitudinally and symmetrically around the middle of the tibialis anterior muscle, 2–3 cm apart. PLMS were scored according to the AASM criteria (Berry et al., 2012) using a validated automated algorithm integrated in the PSG system (Stefani, Heidbreder, Hackner, & Hogl, 2017). Leg movements occurring during a period from 0.5 s preceding a respiratory event to 0.5 s following were not scored, according to AASM criteria (Berry et al., 2012).

### 2.5 Statistics

Statistical analysis was performed using SPSS version 24. Data were tested for normal distribution using the Kolmogorov–Smirnov test. As they were not normal distributed, data are shown as median, range and interquartile range (IQR). For group comparison, non‐parametric tests were applied. The Mann–Whitney *U*‐test was used for the comparison of quantitative variables. Categorical variables were compared with the Fisher's exact test. If more than two groups were compared, χ^2^‐test was applied. Correlations were examined using Spearman's rank correlation coefficient (ρ). A *p*‐value < 0.05 was considered statistically significant.

## 3 RESULTS

### 3.1 Demographic and clinical data

The median age was 59 years, with a median age at onset of RLS symptoms of 50 years, without gender differences. The majority of the patients (52.4%) were not treated for their RLS. Clinical and demographic data stratified by gender are shown in Table 1. Narcolepsy, multiple sclerosis and neurodegenerative diseases (e.g. multiple system atrophy, Parkinson's disease) were not present in our cohort. No history of sleep‐related eating disorder, dystonia, sickle cell disease, post‐traumatic stress disorder, Asperger's syndrome and William's syndrome was found in patients’ records. Other co‐morbidities with possible influence on RLS and PLMS are reported in Table 1.

**Table 1:** Gender distribution of demographic and clinical data

	Women (*n* = 42)	Men (*n* = 42)	*p*‐value
Age, year, median (range) IQR	58.5 (36–81) IQR 52–68.25	59 (36–81) 50–69.5 IQR 50–69.5	0.645
Age of RLS onset, year, median (range) IQR	50 (6–72) IQR 6–72	50 (5–70) IQR 41.25–59.75	0.926
Current augmentation, *n* (%)	2 (4.8)	0 (0)	n.a.
Family history of RLS, *n* (%)	7 (16.7)	5 (12.2)	0.756
Early onset of RLS, *n* (%)	15 (36.6)	17 (41.5)	0.821
Therapy, *n* (%)			
No	22 (52.4)	22 (52.4)	1
α2δ‐ligands	1 (2.4)	1 (2.4)	1
Dopaminergic agents	18 (42.9)	18 (42.9)	1
Dopaminergic agents and α2δ‐ligands	1 (2.4)	1 (2.4)	1
LED, mg, median (range) IQR	35 (8.8–310) IQR 27–130	80 (8.8–227) IQR 23.5–100	0.781
LED per kg, median (range) IQR	0.43 (0.11–5.85) IQR 0.37–2.32	0.87 (0.1–1.92) IQR 0.29–1.31	0.806
Co‐morbidities, *n* (%)			
SRBD	23 (54.8)	36 (85.7)	0.004
Arterial hypertension	16 (44.4)	20 (47.6)	0.509
Radicular pain syndrome	10 (23.8)	6 (14.3)	0.405
Alcohol dependency	1 (2.4)	5 (11.9)	0.202
Congestive heart failure	0 (0)	4 (9.5)	n.a.
RBD	0 (0)	2 (2.4)	n.a.
Renal failure	2 (4.8)	0 (0)	n.a.
Spinal cord injury	1 (2.4)	0 (0)	n.a.

IQR, interquartile range; LED, levodopa equivalent dose; RBD, REM sleep behaviour disorder; RLS, restless legs syndrome; SRBD, sleep‐related breathing disorder.

**Figure 1:**
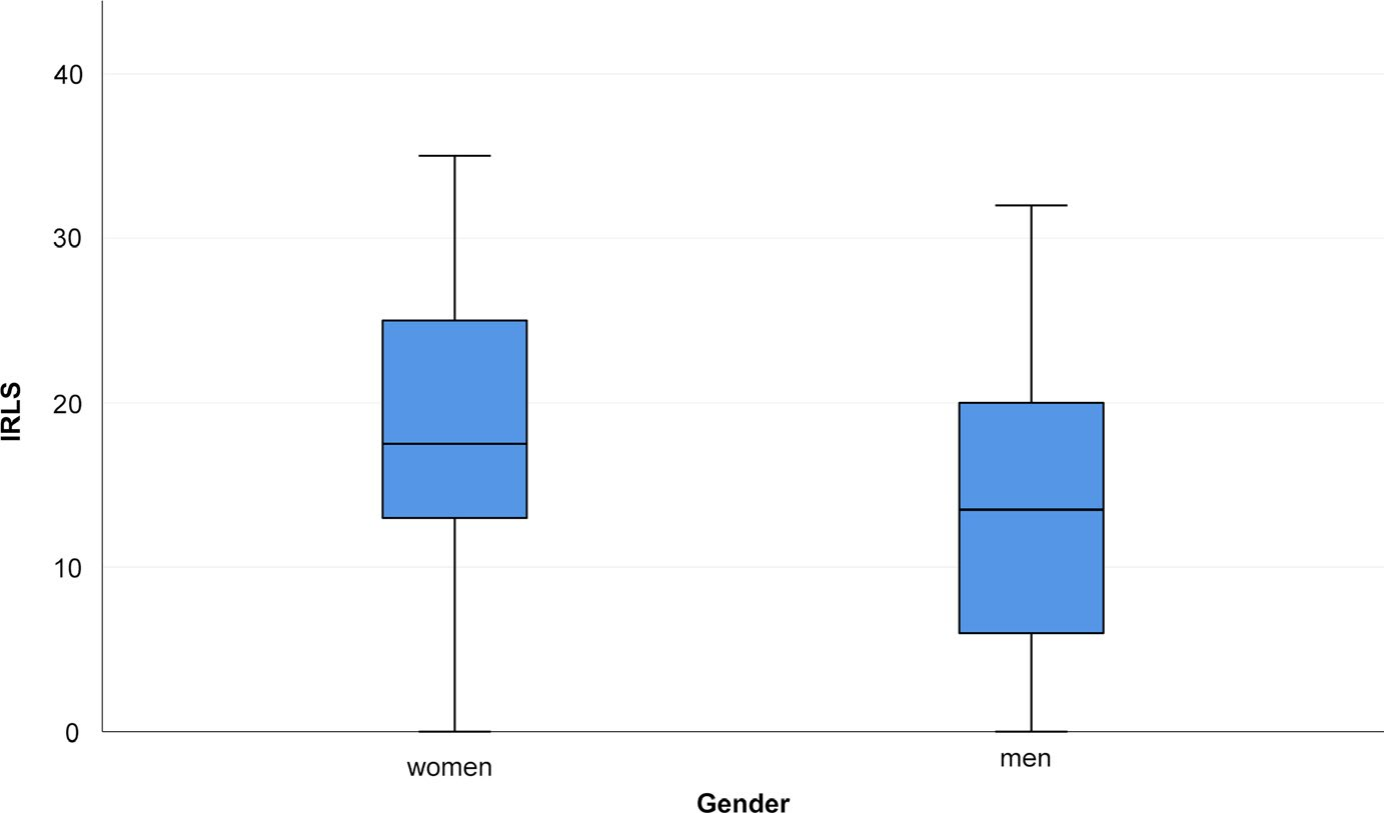
IRLS in women and men. IRLS, International Restless Legs Syndrome Study Group Rating Scale

**Figure 2:**
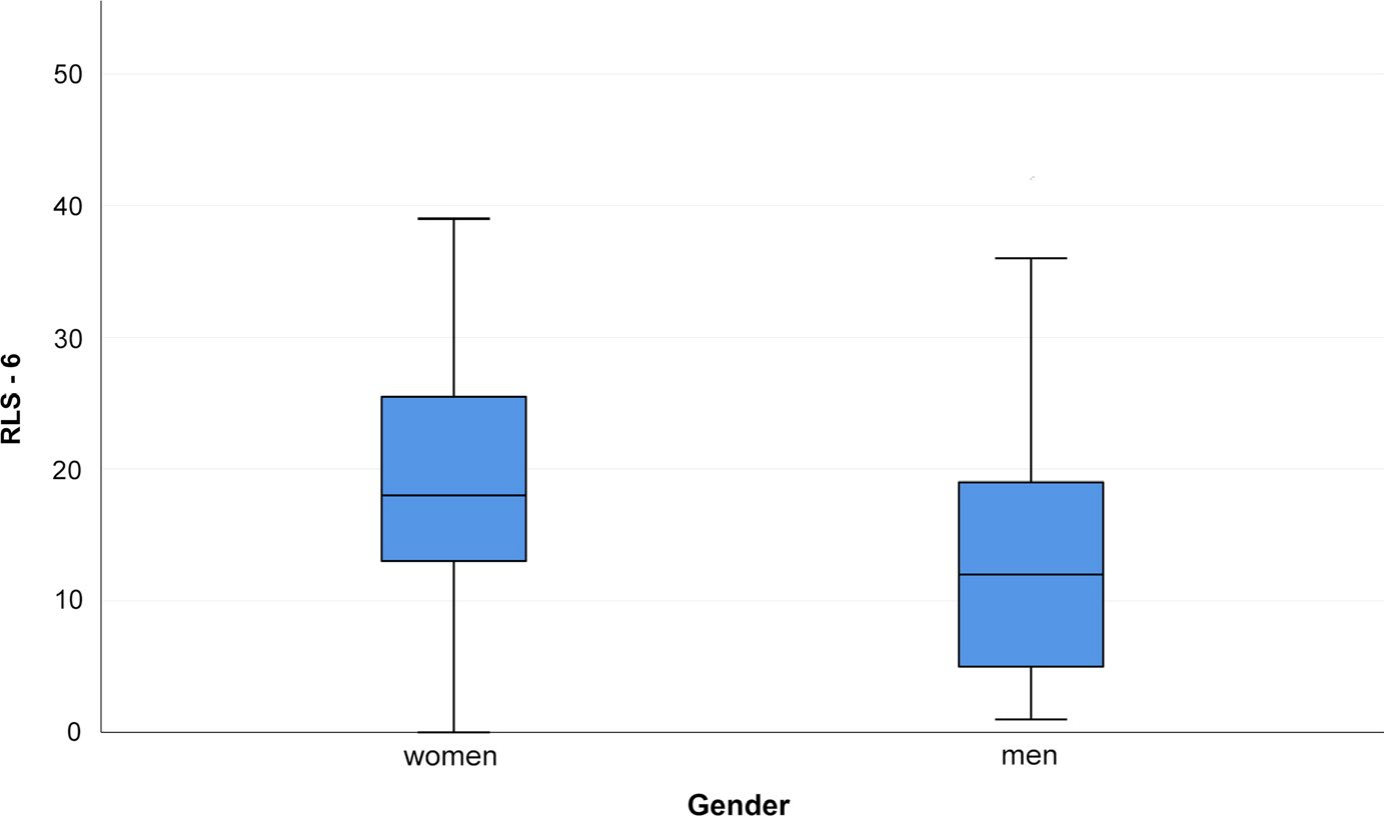
RLS‐6 in women and men. RLS‐6, Restless Legs Syndrome 6 Scale

IRLS was higher in women than in men (median [range] IQR in women versus men: 17.5 [0–35], IQR 12.5–25 versus 13.5 [0–32] IQR 5–20, *p* = 0.028), as shown in Figure 1. The same was true for the total RLS‐6 (median [range] IQR in women versus men: 18 [0–39] IQR 13–25.75 versus 12 [1–42] IQR 5–21.5, *p* = 0.014), shown in Figure 2. When looking into the single RLS‐6 questions, only the first one (RLS‐6‐1) showed a statistically significant difference (median [range] IQR in women versus men: 6 [2–10] IQR 5–8 versus 5 [0–10] IQR 1–6.5, *p* = 0.002). The other items showed no gender differences (*p* > 0.05). CGI was higher in women than in men (median [range] IQR: 4 [1–6], IQR 3–4 versus 3 [1–5] IQR 2–4, *p* = 0.047).

IRLS was higher in patients with early onset of RLS than in late‐onset patients (median [range] IQR: 19 [0–33] IQR 14.75–26 versus 13 [0–36] IQR 4–19, *p* = 0.034), but RLS‐6, RLS‐6‐1 and CGI did not differ (all *p* > 0.05). After gender stratification, the difference in IRLS between early‐ and late‐onset patients was not significant anymore (*p* > 0.05).

IRLS, RLS‐6 and CGI did not differ between patients with a family history for RLS and those without (all *p* > 0.05). Also, when analysing women and men separately, IRLS, RLS‐6 and CGI showed no differences (*p* > 0.05). RLS severity as measured by scales did not differ between the therapy groups (*p* > 0.05).

### 3.2 Laboratory data

For the analysis of laboratory parameters, one woman and four men were excluded because of transferrin saturation ≥ 45%. Two more men, one with serum ferritin ≥ 400 μg L^−1^ and one with both elevated transferrin saturation and serum ferritin, were excluded. The remaining 41 women and 36 men were included in the analysis of laboratory parameters. Serum iron levels did not differ (median [range] IQR in women versus men [μmol L^−1^]: 16.5 [5.6–28.1], IQR 13.85–19.65 versus 17.4 [8.7–26.1], IQR 11.85–20.83, *p* = 1.0). However, women had lower serum ferritin levels than men (median [range] IQR [μg L^−1^]: 74 [9–346], IQR 39–112.5 versus 167 [15–389], IQR 87–246.25, *p* < 0.001). Twenty‐two women and eight men (53.7% versus 22.2%, *p* = 0.006) had ferritin values below 75 μg L^−1^. Serum ferritin levels did not correlate with IRLS, RLS‐6 or CGI (IRLS: ρ = 0.054, *p* = 0.638 in the overall sample, ρ = 0.099, *p* = 0.538 in women, ρ = 0.252, *p* = 0.138 in men; RLS‐6: ρ = 0.018, *p* = 0.878 in the overall sample, ρ = 0.037, *p* = 0.825 in women, ρ = 0.142, *p* = 0.416 in men; CGI: ρ = 0.043, *p* = 0.738 in the overall sample, ρ = 0.074, *p* = 0.667 in women, ρ = 0.236, *p* = 0.247 in men).

IRLS, RLS‐6 and CGI did not differ significantly in patients with ferritin levels below or above 75 μg L^−1^ (IRLS: 15 [0–31] IQR 10–21.5 versus 16 [0–36] IQR 11–25, *p* = 0.71 in the overall sample; 16 [0–31] IQR 13.25–24 versus 21.5 [0–35] IQR 13.25–28, *p* = 0.538 in women; 9 [0–20] IQR 0–15 versus 15 [0–36] IQR 6–20, *p* = 0.251 in men; RLS‐6: 16 [0–35] IQR 10–21 versus 15 [0–42], IQR 9–26, *p* = 0.973 in the overall sample; 16.5 [0–35] IQR 13–23.5 versus 19 [6–39] IQR 10–27, *p* = 0.856 in women; 2 [4–25] IQR 5–18 versus 13 [0–42] IQR 8–25, *p* = 0.384 in men; CGI; 3 [1–5] IQR 3–4 versus 3 [1–6] IQR 3–4, *p* = 0.994 in the overall sample; 4 [1–5] IQR 3–4 versus 4 [2–6] IQR 3–4, *p* = 0.731 in women; 2.5 [2–4] IQR 2–3.75 versus 3 [1–5] IQR 2–4, *p* = 0.471 in men; results reported as median [range] IQR).

No gender differences were found in transferrin, transferrin saturation and soluble transferrin receptor (transferrin [mg dl^−1^]: 259 [214–415] IQR 229.5–287.5 versus 252.5 [185–368] IQR 234.25–293.5, *p* = 0.606; transferrin saturation [%]: 25 [8–44] IQR 20–31.5 versus 26 [12–39] IQR 18.25–32.75, *p* = 0.902; soluble transferrin receptor [g L^−1^]: 2.7 [2.1–4] IQR 2.4–3.23 versus 2.3 [1.9–5.8] IQR 2.05–4, *p* = 0.422; results reported as median [range] IQR in women versus men).

No differences in iron parameters were present among patients with or without a confirmed family history of RLS (*p* > 0.05), as well as among patients with early or late onset of disease (*p* > 0.05), or in the therapy groups (*p* > 0.05).

### 3.3 Polysomnographic data

Regarding PSG sleep variables, women showed lower percentages of N1 sleep in total sleep time (TST) and higher percentages of N3 sleep in TST. PLMS indices during night I and night II were significantly higher in men than in women (Tables 2 and 3).

**Table 2:** Polysomnographic parameters in women and men – night I

	Women (*n* = 42)	Men (*n* = 42)	*p*‐value
Sleep efficacy, %, median (range) IQR	80.6 (44.7–95.2) IQR 71.2–86.78	82.4 (15.1–98.1) IQR 70.23–89.73	0.537
Sleep latency N1, min, median (range) IQR	13.45 (0.5–232.5) IQR 6.98–21.7	10.3 (1–157) IQR 4.23–18.93	0.212
REM sleep latency, min, median (range) IQR	129.5 (54.5–460) IQR 83.75–223–13	133.5 (13.5–497.5) IQR 78.5–201.25	0.707
Wake after sleep onset, min, median (range) IQR	69 (12.5–256) IQR 43.25–112.75	57 (6–327.5) IQR 57–118.75	0.539
N1 sleep, %, median (range) IQR	12.5 (4.4–25.3) IQR 9.05–16.2	16.5 (5.8–98.1) IQR 11.7–24.48	**0.002**
N2 sleep, %, median (range) IQR	47.2 (28.2–78.9) IQR 39–52–58	41–85 (0–72.9) IQR 34.33–55.13	0.316
N3 sleep, %, median (range) IQR	9 (0–22.5) IQR 1.93–14.13	1.8 (0–22.9) IQR 0–6.73	**0.002**
AHI, *n* per hr, median (range) IQR	3.45 (0–51.8) IQR 1.35–10.15	6.55 (0–91.7) IQR 0.88–18.08	0.220
REM sleep, %, median (range) IQR	13.75 (0–26.6) IQR 8.1–18.28	15 (0–32.3) 9.13–19.2	0.730
ODI > 4%, *n* per hr, median (range) IQR	3.25 (0–49.3) IQR 0.9–9.53	6.1 (0–86.9) IQR 0.7–21.18	0.087
PLMS index, *n* per hr, median (range) IQR	11.4 (0–62.5) IQR 5.28–36.03	40 (0–154) IQR 15.5–62	**0.004**
IMI in TST, s, median (range) IQR	30.1 (18.8–44.3) IQR 26.7–33.53	30.85 (19.9–42.2) IQR 24.68–36.63	0.614
PLMW index, *n* per hr, median (range) IQR	0 (0–138.3) IQR 0–0.25	0 (0–336.7) IQR 0–44,6	0.325
IMI in wakefulness, s, median (range) IQR	22.75 (0–37.8) IQR 20.03–30.08	22.9 (10.2–31.5) IQR 19.1–27.3	0.916

AHI, apnea–hypopnea index; IMI, inter‐movement interval; IQR, interquartile range; ODI, oxygen desaturation index; PLMS, periodic leg movements during sleep; PLMW, periodic leg movements during wakefulness; REM, rapid eye movement; TST, total sleep time. Bold values indicates *p* < 0.05.

**Table 3:** Polysomnographic parameters in women and men – night II

	Women (*n* = 42)	Men (*n* = 42)	*p*‐value
Sleep efficacy, %, median (range) IQR	81 (44.7–97.4) IQR 69–81.5	80.7 (54.2–98.1) IQR 72.1–89.8	0.525
Sleep latency N1, min, median (range) IQR	10.8 (0.5–232.5) IQR 4.48–23.88	8.7 (1–157) IQR 3.93–21.10	0.511
REM sleep latency, min, median (range) IQR	128.5 (37.5–460) IQR 78.88–195.38	116 (25.5–371) IQR 69.25–175.88	0.338
Wake after sleep onset, min, median (range) IQR	64.5 (8–256) IQR 41.63–108.75	57 (6–161) IQR 35.75–93.5	0.270
N1 sleep, %, median (range) IQR	11.05 (4.4–25.3) IQR 8.45–14.08	14 (5.8–59.6) IQR 10.5–18.7	**0.009**
N2 sleep, %, median (range) IQR	46.7 (28.2–78.9) IQR 38.05–52.5	47.6 (11.2–72.9) IQR 37.9–54.28	0.674
N3 sleep, %, median (range) IQR	11.05 (0–22) IQR 2.28–17.73	0.5 (0–24) IQR 0–6.88	**< 0.001**
REM sleep, %, median (range) IQR	16.5 (0–26.6) IQR 8.68–20.05	18.45 (0–34.8) IQR 14.18–23.43	0.085
AHI, *n* per hr, median (range) IQR	2.3 (0–26.7) IQR 0–4.53	2.85 (0–30.2) IQR 0.78–7.8	0.398
ODI > 4%, *n* per hr, median (range) IQR	2 (0–22.8) IQR 0.58–4.05	3 (0–20–9) IQR 0.5–6.45	0.204
PLMS index, *n* per hr, median (range) IQR	12.6 (0–58.5) IQR 5.35–33.75	40 (0.5–208) IQR 15–75.8	**0.002**
IMI in TST, s, median (range) IQR	29.45 (18.8–44.3) IQR 24.68–33.6	30.8 (11.2–42.2) IQR 23.3–34.8	0.919
PLMW index, *n* per hr, median (range) IQR	0 (0–108.6) IQR 0–0.5	0 (0–138.7) IQR 0–30.88	0.424
IMI in wakefulness, s, median (range) IQR	23.2 (14.6–44.9) IQR 20.65–35	22.7 (18–33.1) 19.2–25.6	0.774

AHI, apnea–hypopnea index; IMI, inter‐movement interval; IQR, interquartile range; ODI, oxygen desaturation index; PLMS, periodic leg movements during sleep; PLMW, periodic leg movements during wakefulness; REM, rapid eye movement; TST, total sleep time. Bold values indicates *p* < 0.05.

No correlation between PLMS indices and IRLS was found, even after gender stratification (overall sample, night I: ρ = 0.012, *p* = 0.917; night II: ρ = 0.012, *p* = 0.913; women, night I: ρ = 0.03, *p* = 0.855; night II: ρ = 0.059, *p* = 0.717; men, night I: ρ = 0.07, *p* = 0.666; night II: ρ = 0.116, *p* = 0.468). PLMS indices did not correlate with ferritin in the whole sample (night I: ρ = −0.001, *p* = 0.99; night II: ρ = −0.1, *p* = 0.93) and in women (night I: ρ = 0.006, *p* = 0.971; night II: ρ = 0.053, *p* = 0.751). In men only, PLMS indices correlated with serum ferritin values (night I: ρ = −0.37, *p* = 0.029; night II: ρ = −0.411, *p* = 0.014). PLMS indices showed no significant differences between the therapy groups (overall sample, night I: *p* = 0.369, night II: *p* = 0.516; women, night I: *p* = 0.2347, night II: *p* = 0.364; men, night I: *p* = 0.671, night II: *p* = 0.438). PLMS indices and LED did not correlate (all patients with dopaminergic therapy, night I: ρ = 0.007, *p* = 0.95; night II: ρ = 0.046, *p* = 0.691; women, night I: ρ = −0.159, *p* = 0.326; night II: ρ = −0.211, *p* = 0.192; men, night I: ρ = 0.174, *p* = 0.295; night II: ρ = 0.299, *p* = 0.68). No differences in PLMS indices were present among patients with or without a confirmed family history of RLS, as well as among patients with early or late onset of disease, also after gender stratification (all *p* > 0.05). PLMS indices and apnea–hypopnea indices (AHI) correlated in the overall sample (night I: ρ = 0.349, *p* = 0.001; night II: ρ = 0.305, *p* = 0.006). When analysing women and men separately, a correlation was found in women (night I: ρ = 0.409, *p* = 0.009; night II: ρ = 0.39, *p* = 0.013), but not in men (night I: ρ = 0.2, *p* = 0.209; night II: ρ = 0.238, *p* = 0.134).

## 4 DISCUSSION

This is to our knowledge the first study to address gender differences in RLS phenotypes by simultaneously assessing clinical, laboratory and PSG parameters. We found higher IRLS, RLS‐6 and CGI scores in women, while ferritin levels and PLMS indices were higher in men.

In our sample, RLS symptoms, as subjectively measured using the IRLS and RLS‐6, and as evaluated by the examiner with the CGI, were more severe in women than in men. This may suggest that the same RLS symptom burden is felt differently in women and men, with women considering the same RLS symptoms as affecting daily life more than men. Alternatively, it can be hypothesized that women are more aware of RLS symptoms compared with men. We cannot rule out that RLS severity in women was already higher when they came to the Sleep Disorders Unit, because women are more likely referred to PSG because of RLS, whilst men are more frequently referred for sleep‐related breathing disorders (SRBD), with RLS as an incidental finding (Auer et al., 2018). RLS severity did not differ between patients with and without a confirmed family history of RLS. A positive family history of RLS was less frequent in our RLS sample than expected (14.5% in our sample versus 60% in the literature; Winkelmann et al., 2007), probably because we only rated family history as positive if the diagnosis was not only reported but confirmed by a physician in a family member. Slight differences in symptoms severity may be difficult to detect using the CGI, due to the limited answer options (from 1 to 7), while the IRLS and nowadays also the self‐administered IRLS (sIRLS; Sharon et al., 2018) are more fine‐tuned instruments, with a range from 0 to 40 points. Despite these numerical differences, mean RLS severity is classified as moderate in both women and men based on IRLS.

Women presented with lower ferritin values compared with men, in line with data from the literature (Koziol, Ho, Felitti, & Beutler, 2001; Zacharski, Ornstein, Woloshin, & Schwartz, 2000). Moreover, almost half of the women in our cohort presented with ferritin values ≤ 75 μg L^−1^. As an inverse correlation between IRLS and serum ferritin has been reported in studies from others and our own group (Frauscher et al., 2009; O'Keeffe, Gavin, & Lavan, 1994), we hypothesized that iron deficiency in women could be a possible explanation for the higher RLS severity. However, in our sample no correlation between RLS severity and ferritin was found, opposing former findings from the Innsbruck RLS cohort (Frauscher et al., 2009). Our sample size might have been too small to reproduce previous findings. Given the fact that iron deficiency in RLS does probably not take place on the systemic or cytosolic but on the mitochondrial level (Haschka et al., 2018), serum ferritin may not be the ideal biomarker for iron stores in RLS.

Women had significantly less N1 sleep and more N3 sleep than men. No gender differences in sleep stage distribution were described in healthy sleepers (Mitterling et al., 2015), but our findings are congruent with previous data in patients with RLS, reporting higher percentages of N1 sleep and lower percentages of slow‐wave sleep in men (Nicolas et al., 1999). In our RLS sample, a connection to the higher PLMS indices and subsequent micro‐arousals followed by N1 sleep in men could be speculated. Although women showed more N3 sleep, they were less satisfied with their sleep than men, as reflected in the RLS‐6‐1 scale, indicating that in our sample women experienced their RLS symptoms as more disturbing than men.

The PLMS indices were higher in male compared with female patients with RLS. Similar results have been previously reported in untreated RLS patients (Montplaisir et al., 1997; Shin et al., 2016). In our study, we did not observe any correlation between PLMS indices and ferritin values. An association between PLM and low ferritin in probable RLS patients was reported in the Wisconsin sleep cohort. In this study, however, participants were only stratified based on a questionnaire, which did not address all diagnostic criteria and did not exclude mimics (Li et al., 2015). In patients with RLS diagnosed according to current criteria through a clinical interview by an expert in sleep medicine, with exclusion of mimics (Allen et al., 2014), no association between iron levels and PLMS was found (Heidbreder et al., 2017). In this study, patients were also selected from the Innsbruck RLS database. However, we can exclude a selection bias as there was no overlap in patients included in these two studies. Dopaminergic agents have been reported to lower PLMS indices in patients with RLS (Salminen & Winkelmann, 2018). We can rule out that treatment differences, i.e. more women treated with dopamine agonists or with higher dosage compared with men, could have biased our results, as patients were matched not only for age but also for therapy. Moreover, in the group of patients treated with dopaminergic drugs neither the LED nor the LED per kg differed between women and men. More men than women suffered from SRBD, and correlations between PLMS indices and AHI were found in our sample, so it could appear that high PLMS indices in men were secondary to high AHI. Interestingly, after gender stratification this correlation was only present in women, indicating that the relationship only existed in subjects with lower PLMS indices. Moreover, we compared PLMS indices not only in the first night, but also in a subsequent night with adequate sleep apnea treatment. The gender differences in PLMS indices persisted, whereas AHI in both nights did not differ between women and men. Furthermore, when scoring PLMS indices, respiratory‐related leg movements were excluded. The frequency of most co‐morbidities with a possible influence on PLMS did not differ between men and women. Two women with renal failure reported RLS symptoms many years before the renal failure, as did one patient with a spinal cord injury. In two men, RBD was detected by PSG after they initially presented because of RLS. We repeated the comparison of PLMS indices excluding these patients with RBD, and gender difference was still present (*p* = 0.009 for night I, and *p* = 0.005 for night II). Because of the retrospective data acquisition, we cannot rule out that congestive heart failure preceded RLS in the four men with this condition. Nevertheless, we did not exclude them from the analysis as current literature data show that the association with RLS should be limited to a few diseases, such as iron deficiency anaemia and uraemia (Trenkwalder et al., 2018). Anyway, even after additional exclusion of these four patients, PLMS indices were higher in men than in women (*p* = 0.004 for night I, and *p* = 0.002 for night II).

One of the major strengths of our study is the well‐defined sample of patients with RLS, which includes strictly matched patients (matched not only for age, but also for therapy). In addition to clinical and laboratory data, this study also analysed PSG parameters in all patients. The effect of other medications with a possible influence on RLS and PLMS was ruled out by the exclusion criteria. The main limitations are retrospective data analysis, the limited sample size, and that we cannot completely rule out a relationship between AHI and PLMS indices.

In summary, our data show higher RLS severity but lower PLMS indices in women, as compared with men. A possible explanation for these results may be a gender difference in clinical manifestations of RLS, with women experiencing predominantly sensory symptoms, reflected by higher IRLS severity scores, and men showing predominantly motor symptoms, represented by higher PLMS indices. This hypothesis should be further investigated in prospective studies with a larger sample size.

## CONFLICT OF INTERESTS

The authors have no conflict of interest related to this work to report.

## AUTHOR CONTRIBUTIONS

Study design: A. S., B. H.; data acquisition: E. H., A. S.; data analysis: E. H., A. S.; interpretation of results: A. S., B. H.; first drafting of the manuscript: E. H.; critical revision of the manuscript: A. S., B. H., M. H., G. W. All authors approved the final version of the manuscript.
